# Apoptosis signal-regulating kinase 1 promotes inflammation in senescence and aging

**DOI:** 10.1038/s42003-024-06386-0

**Published:** 2024-06-05

**Authors:** Takeru Odawara, Shota Yamauchi, Hidenori Ichijo

**Affiliations:** 1 Laboratory of Cell Signaling, Graduate School of Pharmaceutical Sciences, The University of Tokyo, Tokyo, Japan; 2 Cell Signaling and Stress Responses Laboratory, Advanced Research Institute (ARIS), Tokyo Medical and Dental University, Tokyo, Japan

**Keywords:** Senescence, Ageing, Stress signalling, Inflammation

## Abstract

Cellular senescence is a stress-induced, permanent cell cycle arrest involved in tumor suppression and aging. Senescent cells secrete bioactive molecules such as pro-inflammatory cytokines and chemokines. This senescence-associated secretory phenotype (SASP) has been implicated in immune-mediated elimination of senescent cells and age-associated chronic inflammation. However, the mechanisms regulating the SASP are incompletely understood. Here, we show that the stress-responsive kinase apoptosis signal-regulating kinase 1 (ASK1) promotes inflammation in senescence and aging. ASK1 is activated during senescence and increases the expression of pro-inflammatory cytokines and chemokines by activating p38, a kinase critical for the SASP. ASK1-deficient mice show impaired elimination of oncogene-induced senescent cells and an increased rate of tumorigenesis. Furthermore, ASK1 deficiency prevents age-associated p38 activation and inflammation and attenuates glomerulosclerosis. Our results suggest that ASK1 is a driver of the SASP and age-associated chronic inflammation and represents a potential therapeutic target for age-related diseases.

## Abstract (web summary)

Apoptosis signal-regulating kinase 1 (ASK1) drives the senescence-associated secretory phenotype and contributes to immune-mediated clearance of precancerous senescent cells and age-related chronic inflammation.

## Introduction

Cellular senescence is a state of persistent cell cycle arrest that is induced by various stresses, such as telomere shortening, oncogene activation, and DNA damage^1^. These stresses initially activate the transcription factor p53, which increases the expression of the cyclin-dependent kinase (CDK) inhibitor p21 (ref. ^2^). If the stress persists, the expression of p16, also a CDK inhibitor, is increased to maintain the cell cycle arrest. Senescence has long been known as a tumor suppressive mechanism that prevents the proliferation of potentially tumorigenic cells^1^. In addition, recent studies have shown that senescence occurs in various tissues over time and contributes to aging^2^.

Senescent cells exert a variety of physiological functions through the secretion of a complex combination of pro-inflammatory cytokines (e.g., IL-1β, IL-6), chemokines (e.g., IL-8, CCL2), and growth factors, termed the senescence-associated secretory phenotype (SASP)^3^. The SASP can be beneficial or detrimental depending on the biological context. For example, the SASP is beneficial in that it mediates the recruitment of immune cells to eliminate senescent cells^4^. This elimination is called senescence surveillance and prevents oncogene-induced senescent cells from developing into tumors^4^. In a mouse model of hepatocyte senescence, senescence surveillance was reported to involve macrophage recruitment via CCL2 (ref. ^5^). On the other hand, the SASP is detrimental through the contribution to age-associated chronic inflammation^6^. Clearance of senescent cells from tissues reduces the expression levels of pro-inflammatory cytokines and extends lifespan in naturally or accelerated aging mice^7,8^. Conversely, transplantation of senescent preadipocytes into young mice leads to physical dysfunction and shortened lifespan^9^.

Chronic inflammation is a hallmark of aging^10^. Elevated levels of pro-inflammatory cytokines in the elderly are associated with the risk of age-related diseases such as atherosclerosis, diabetes, and neurodegenerative disorders^11–13^. Blockade of TNF-α or knockout of the inflammasome protein NLRP3 suppresses inflammation in aged mice and ameliorates age-related pathologies such as cardiac hypertrophy and decline in motor and cognitive function^14–17^. It has been reported that approximately 40% of the proteins that are increased in aging plasma are associated with the SASP^18^. This implies the potential of drugs that suppress the SASP, called senomorphics, as a treatment for age-related diseases^19^. Therefore, the molecular mechanisms regulating the SASP have been the subject of intense research in recent years.

Previous studies have identified several SASP drivers such as NF-κB, C/EBP-β, and p38 mitogen-activated protein kinase (MAPK)^20–22^. p38 promotes SASP factor transcription and, at the post-transcriptional level, stabilizes SASP factor mRNAs^22,23^. The importance of p38 in the SASP is supported by the finding that inhibition of p38 reduces the tumor-promoting activities of senescent fibroblasts in the tumor microenvironment^23^. In the MAPK cascade, MAP3K phosphorylates and activates MAP2K, which in turn phosphorylates and activates MAPK. The MAP3Ks involved in p38 activation during senescence remain unclear.

In this study, we report that the stress-responsive MAP3K apoptosis signal-regulating kinase 1 (ASK1) activates p38 during senescence and potentiates the expression of SASP factors. Using a mouse model, we show that ASK1 contributes to the elimination of precancerous senescent hepatocytes through macrophage recruitment to the liver. Moreover, we reveal that the ASK1-p38 pathway is also activated during aging. In aged mice, ASK1 increases the expression of pro-inflammatory cytokines and promotes glomerulosclerosis. Our findings demonstrate that ASK1 activation is crucial for inflammation associated with senescence and aging.

## Results

### ASK1 promotes the SASP by activating p38

**Fig. 1:**
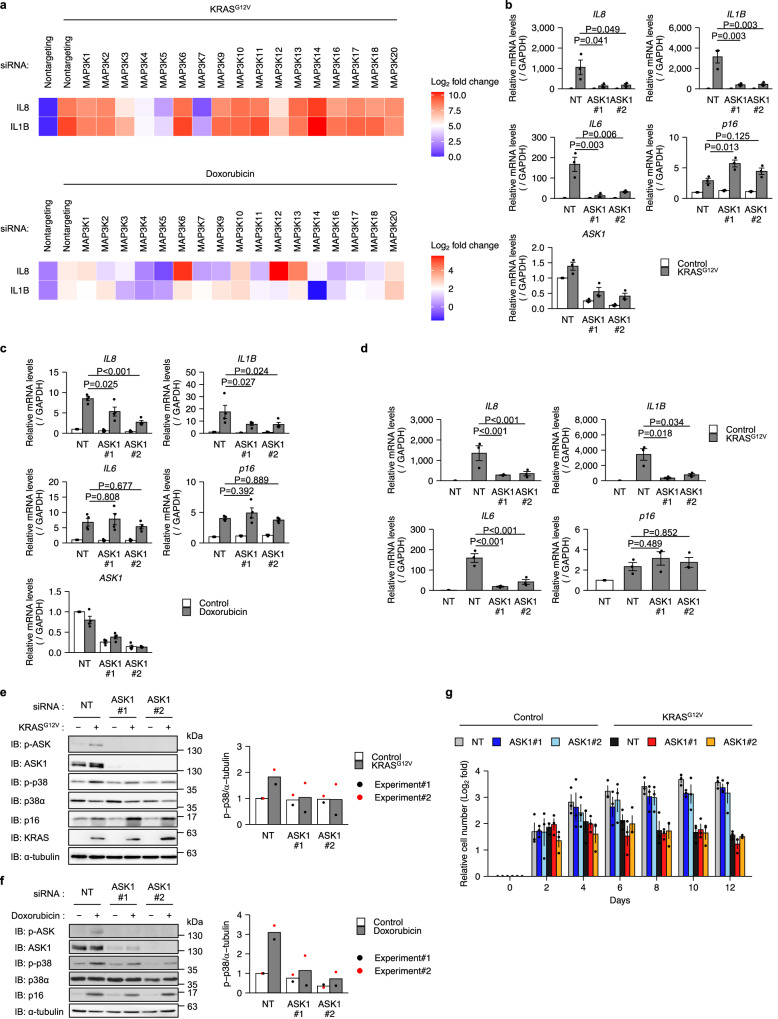
ASK1 promotes the SASP by activating p38. **a** Heatmap of the mRNA levels for IL8 and IL1B. Cells were transfected with non-targeting (NT) or indicated MAP3K siRNAs and treated with 4OHT (IMR-90 ER-KRAS) or doxorubicin (IMR-90) for 12 days. The mRNA levels were analyzed by qPCR. *n* = 3 (top), 2 (bottom) independent experiments. Data are mean. **b** qPCR analysis of IMR-90 ER-KRAS cells transfected with NT and ASK1 siRNAs and treated with 4OHT for 12 days. *n* = 3 independent experiments. **c** qPCR analysis of IMR-90 cells transfected with NT and ASK1 siRNAs and treated with doxorubicin for 12 days. *n* = 4 independent experiments. **d** qPCR analysis of IMR-90 ER-KRAS cells transfected with NT and ASK1 siRNAs at day 8 after 4OHT treatment. *n* = 3 independent experiments. Bars represent mean ± s.e.m. **b**–**d** Immunoblot analysis of indicated cells transfected with NT and ASK1 siRNAs and treated with 4OHT (**e**) or doxorubicin (**f**) for 12 days. Right graphs depict the quantification of the western blots. Individual values and the mean are presented as points and bars, respectively. *n* = 2 independent experiments (**e**, **f**). **g** Growth of IMR-90 ER-KRAS cells transfected with NT and ASK1 siRNAs and treated with 4OHT or DMSO. *n* = 3 independent experiments. Data are mean ± s.e.m. Statistical analysis was performed using two-way ANOVA followed by Dunnett’s multiple comparison test.

p38 MAPK is critical for the SASP, but its upstream MAP3Ks remain unclear. We performed an siRNA screen for MAP3Ks involved in the SASP. We used IMR-90 primary human fibroblasts in which KRAS^G12V^ expression is inducible with 4-hydroxy Tamoxifen (4OHT) (hereafter IMR-90 ER-KRAS), a well-characterized model of oncogene-induced senescence (OIS)^24^. We also induced senescence by treating IMR-90 cells with the DNA-damaging agent doxorubicin. Among the MAP3Ks expressed in IMR-90 cells, knockdown of MAP3K5 (ASK1), MAP3K7 (TAK1) and, to a lesser extent, MAP3K4 (MTK1) suppressed senescence-associated upregulation of IL-8 and IL-1β in both OIS and DNA damage-induced senescence (DIS) (Fig. 1a).

ASK1 is a MAP3K that activates p38 and JNK in stress and immune responses^25^. We and others have reported that ASK1 is involved in diverse diseases such as cardiac hypertrophy^26^, Parkinson’s disease^27^, and osteoarthritis^28^. Notably, these diseases have also been reported to be associated with senescence^29–31^. Therefore, we set out to investigate the role of ASK1 in senescence. We confirmed by qPCR that knockdown of ASK1 suppressed expression of SASP factors in both OIS and DIS, except for IL-6 in DIS (Fig. 1b, c). Knockdown of ASK1 in already senescent cells also decreased expression of SASP factors (Fig. 1d). Immunoblotting analysis revealed that ASK1 and p38 were activated during OIS and DIS (Fig. 1e, f). p38 activation was suppressed by knockdown of ASK1 (Fig. 1e, f). In contrast, increased expression of p16, a cyclin-dependent kinase inhibitor critical for senescence, was not suppressed by ASK1 knockdown at either the mRNA or protein level (Fig. 1b–f). Knockdown of ASK1 did not significantly affect cell proliferation in 4OHT-treated IMR-90 ER-KRAS cells (Fig. 1g), suggesting that ASK1 is dispensable for the senescence proliferative arrest. Similar to ASK1 knockdown, treatment with SB023580, a p38 inhibitor, suppressed SASP factor expression but not p16 expression (Supplementary Fig. 1). These data indicate that ASK1 promotes the SASP via activation of p38.

### ASK1 mediates macrophage migration through CCL2

**Fig. 2:**
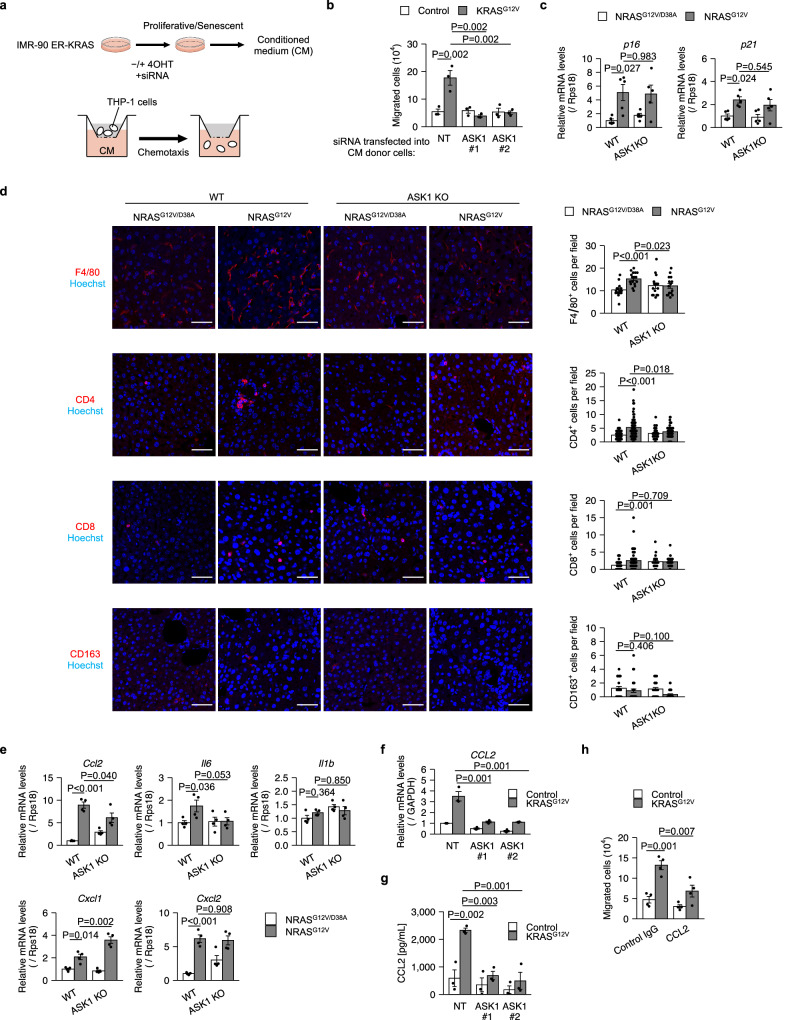
ASK1 mediates macrophage migration through the SASP. **a** Schematic of the migration assay of THP-1 cells with proliferative and senescent IMR-90 ER-KRAS. CM, conditioned media. **b** Migration assay of THP-1 cells. CM was collected from proliferative and senescent IMR-90 ER-KRAS cells transfected with non-targeting (NT) and ASK1 siRNAs. *n* = 3 independent experiments. qPCR analysis (**c**) and representative immunofluorescence images (**d**) of wild-type (WT) and ASK1-knockout (ASK1 KO) mice liver 6 days after hydrodynamic gene delivery. *n* = 5 (**c**), 3 (**d**) mice per group. 5–10 randomly selected fields per mouse were analyzed. Scale bar, 50 μm. **e** qPCR analysis of WT and ASK1 KO mice liver 6 days after hydrodynamic gene delivery. *n* = 4 mice per group. qPCR analysis (**f**) and ELISA (**g**) of IMR-90 ER-KRAS cells transfected with NT and ASK1 siRNAs and treated with 4OHT for 12 days. *n* = 3 independent experiments. **h** Migration assay of THP-1 cells with CM in the presence of CCL2-neutralizing or control IgG antibodies. *n* = 4 independent experiments. Bars represent mean ± s.e.m. **b**–**h**. Statistical analysis was performed using two-way ANOVA followed by Dunnett’s multiple comparison test.

We next sought to explore the role of ASK1 in biological functions of the SASP. Senescent cells attract macrophages and other immune cells through the SASP, thereby promoting their own elimination^4,32,33^. To examine the involvement of ASK1 in macrophage recruitment, we performed a cell migration assay using THP-1 cells (Fig. 2a). Conditioned medium (CM) derived from senescent IMR-90 ER-KRAS cells promoted the migration of THP-1 cells compared to CM derived from proliferative cells (Fig. 2b). This migration was inhibited by knockdown of ASK1 in senescent IMR-90 ER-KRAS cells, suggesting that ASK1 is required for the attraction of macrophage through the SASP in vitro (Fig. 2b).

To investigate whether ASK1 is involved in macrophage recruitment in vivo, we used a mouse model in which hepatocyte senescence is induced by hydrodynamic injection-mediated delivery of an oncogenic NRAS vector (NRAS^G12V^)^4,5,34^. Consistent with previous studies, expression of NRAS^G12V^, but not an inactive mutant of NRAS (NRAS^G12V/D38A^), upregulated the expression of the senescence markers p16 and p21 at day 6 (Fig. 2c). The expression levels of p16 and p21 were comparable between wild-type (WT) and ASK1-knockout (ASK1 KO) mice, indicating that ASK1 does not affect senescence induction (Fig. 2c). In contrast, immunostaining of liver sections revealed that the recruitment of F4/80^+^ macrophages was significantly impaired in ASK1 KO mice (Fig. 2d). Recruitment of CD4^+^ T cells, which help macrophages to eliminate senescent hepatocytes^4^, was also suppressed in ASK1 KO mice (Fig. 2d). Recruitment of CD8^+^ T cells was not significantly suppressed (Fig. 2d). NRAS^G12V^ expression did not induce the recruitment of CD163^+^ immunosuppressive macrophages (Fig. 2d). In this experimental model, increased CCL2 expression in NRAS^G12V^-expressing hepatocytes was shown to promote macrophage recruitment^4,5,34^. This increased CCL2 expression was suppressed in ASK1 KO mice (Fig. 2e). Increased IL-6 expression was also suppressed, but this was not statistically significant (Fig. 2e). CXCL1 and CXCL2 expression was increased in both WT and ASK1 KO mice (Fig. 2e). NRAS^G12V^ expression did not increase IL-1β expression (Fig. 2e).

As in the mouse model, ASK1 knockdown suppressed increased CCL2 expression in senescent IMR-90 ER-KRAS cells (Fig. 2f). Enzyme-linked immunosorbent assay (ELISA) showed that ASK1 knockdown also suppressed increased CCL2 levels in CM (Fig. 2g). Furthermore, antibody neutralization of CCL2 in CM attenuated the increased migration of THP-1 cells (Fig. 2h). SB023580 treatment also attenuated the increase in CCL2 expression and THP-1 cell migration (Supplementary Fig. 2). These data indicate that ASK1 contributes to macrophage recruitment, at least in part, by increasing CCL2 expression in senescent cells.

### ASK1 is required for the tumor suppressor function of the SASP

**Fig. 3:**
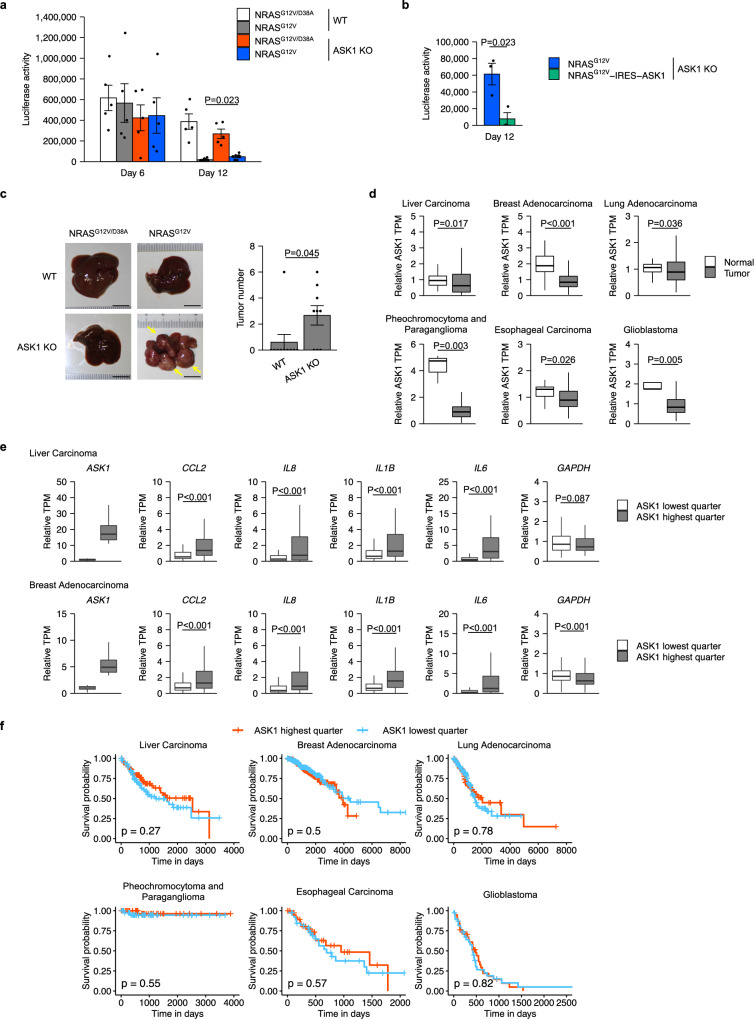
ASK1 is required for the tumor suppressor function of the SASP. **a** Luciferase assay of wild-type (WT) and ASK1-knockout (ASK1 KO) mice liver after hydrodynamic gene delivery. *n* = 5 mice per group. **b** Luciferase assay of ASK1 KO mice liver 12 days after hydrodynamic gene delivery. *n* = 3 mice per group. **c** Representative images of WT and ASK1 KO mice liver 5 months after hydrodynamic gene delivery. *n* = 9 (WT), 10 (ASK1 KO) mice per group. Yellow arrows indicate tumors. Scale bar, 1 cm. Bars represent mean ± s.e.m. (**a**–**c**). **d** RNA-seq analysis of ASK1 expression in normal and tumor tissues. RNA-Seq data were obtained from the TCGA project. Center line, median; box limits, upper and lower quartiles; whiskers, 1.5× interquartile range. **e** RNA-Seq analysis of inflammatory gene expression in tumor tissues with high and low ASK1 expression. RNA-seq data from indicated TCGA project were used for the analysis. **f** Survival curves of the patients with high and low ASK1 expression. Statistical analysis was performed using Wilcoxon rank-sum test (**a**, **c**, **d**, **e**), unpaired two-tailed Student’s *t* test (**b**) and log-rank test (**f**).

We investigated whether ASK1 is involved in the elimination of senescent hepatocytes in the mouse model^4^. To estimate the relative number of NRAS^G12V^-expressing hepatocytes, we measured the activity of luciferase co-expressed with NRAS^G12V^. WT mice showed an NRAS activity-dependent reduction in luciferase activity from day 6 to day 12 after injection of the NRAS^G12V^ vector (Fig. 3a), suggesting that senescent hepatocytes were eliminated during this time period as previously reported^4^. This reduction in luciferase activity was partially but significantly inhibited in ASK1 KO mice (Fig. 3a). Re-expression of ASK1 along with NRAS^G12V^ in hepatocytes of ASK1 KO mice restored the reduction in luciferase activity, suggesting that the elimination of senescent hepatocytes depends on ASK1 expressed in these hepatocytes themselves (Fig. 3b). In this re-expression experiment, we used a vector containing an internal ribosome entry site (IRES) between NRAS^G12V^ and ASK1 so that NRAS^G12V^ and ASK1 were translated from the same mRNA. OIS cells that evade senescence surveillance can develop into tumors^4^. Five months after injection of the NRAS^G12V^ vector, more intrahepatic tumors were observed in ASK1 KO mice than in WT mice (Fig. 3c). These data suggest that ASK1 is associated with the elimination of precancerous senescent cells.

To investigate the role of ASK1 in human cancer, we explored the RNA-seq data in The Cancer Genome Atlas (TCGA). In several cancer types, ASK1 expression was lower in cancer tissues compared to normal tissues (Fig. 3d). In liver and breast cancer, low expression of ASK1 was associated with low expression of pro-inflammatory cytokines and chemokines (Fig. 3e). These data are consistent with the idea that, at least in certain human cancers, ASK1 deficiency impairs senescence surveillance and promotes tumorigenesis. On the other hand, ASK1 expression levels do not appear to be associated with survival in cancer patients, suggesting that once a cancer has formed, ASK1 does not markedly affect its malignancy (Fig. 3f).

### ASK1-p38 pathway promotes age-related inflammation

**Fig. 4:**
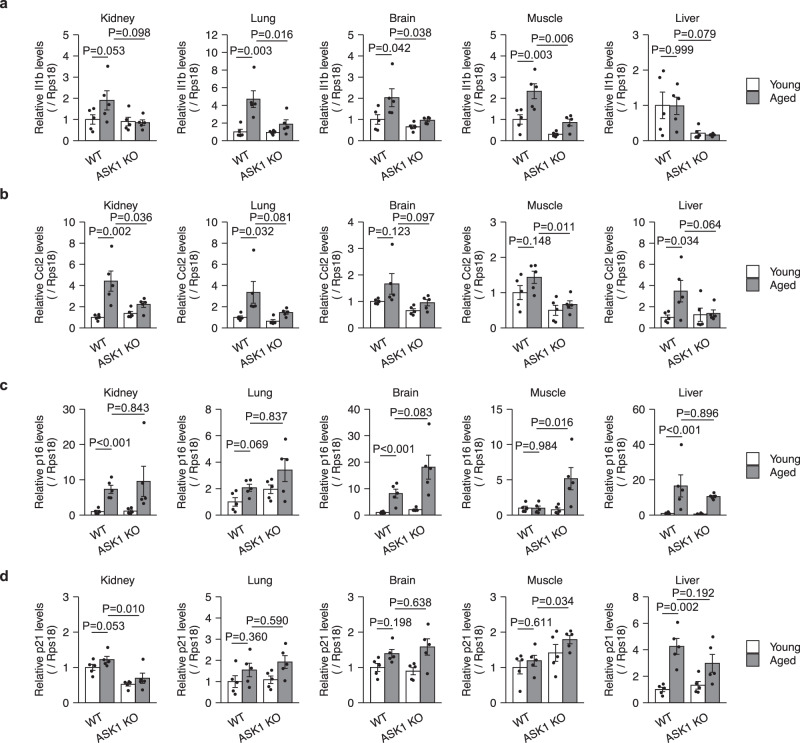
ASK1 is involved in age-related pro-inflammatory gene expression. qPCR analysis of *Il1b* (**a**), *Ccl2* (**b**), *p16* (**c**) and *p21* (**d**) in the indicated tissues from young (3-month-old) and aged (20-month-old), wild-type (WT) and ASK1-knockout (ASK1 KO) mice. *n* = 5 mice per group. Bars represent mean ± s.e.m. (**a**–**d**). Statistical analysis was performed using two-way ANOVA followed by Dunnett’s multiple comparison test.

The SASP is associated with chronic inflammation and diseases during aging^12^. In particular, previous reports indicate that an age-associated increase in serum concentrations of IL-1β contributes to diseases such as type 2 diabetes, Alzheimer’s disease, and motor dysfunction^17,35,36^. CCL2 has also been considered a potential contributor to age-related cognitive dysfunction^37^. In ASK1 KO mice, the age-related increase in IL-1β and CCL2 expression tended to be suppressed in the kidney, lung, brain, and muscle (Fig. 4a, b). In contrast, p16 expression increased with age in both WT and ASK1 KO mice, except in muscle (Fig. 4c). p21 expression increased in the liver of WT and ASK1 KO mice (Fig. 4d).

**Fig. 5:**
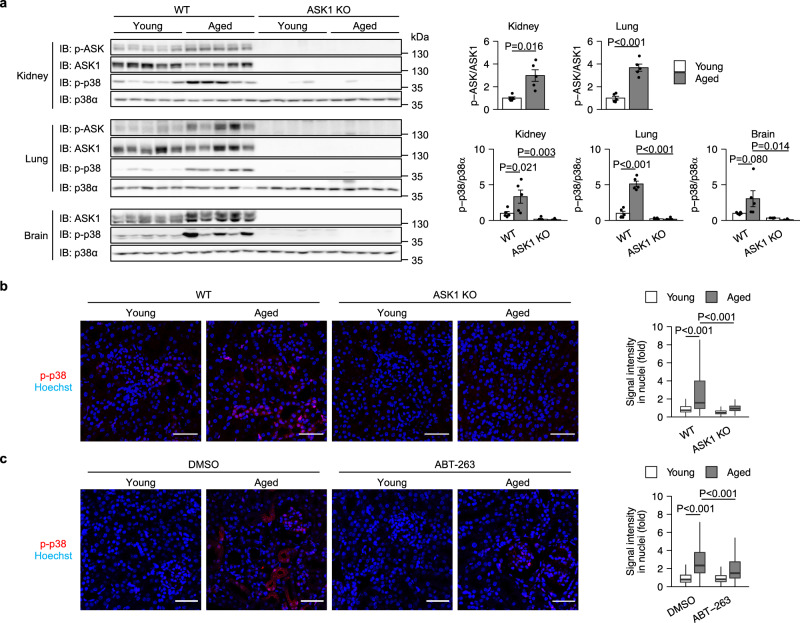
ASK1-p38 pathway is activated with age. **a** Immunoblot analysis (left) and band quantification (right) of the indicated tissues from WT and ASK1 KO mice. *n* = 5 mice per group. Bars represent mean ± s.e.m. **b** Representative immunofluorescence images (left) and quantification (right) of kidneys from WT and ASK1 KO mice. Scale bar, 50 μm. *n* = 3 mice per group. 10 randomly selected fields per mouse were analyzed. Center line, median; box limits, upper and lower quartiles; whiskers, 1.5× interquartile range. **c** Representative immunofluorescence images (left) and quantification of phospho-p38 signal (right) of kidneys from young and aged WT mice treated with DMSO or ABT-263. 10 randomly selected fields per mouse were analyzed. Scale bar, 50 μm. *n* = 3 mice per group. Statistical analysis was performed using two-way ANOVA followed by Dunnett’s multiple comparison test.

ASK1 has been suggested to be activated in the Klotho KO model of accelerated aging^38^. We found that ASK1 and p38 were activated in the kidney and lung of aged mice (Fig. 5a, b). Furthermore, p38 activation was suppressed in ASK1 KO mice (Fig. 5a, b). Although ASK1 activity could not be evaluated in the brain, age-associated p38 activation was suppressed in ASK1 KO mice (Fig. 5a). These data indicate that the ASK1-p38 pathway is activated during aging and contributes to pro-inflammatory cytokine expression.

Senescent cells that accumulate in aged kidney tissue cause age-associated renal inflammation and dysfunction^8^. We asked whether cellular senescence is required for the activation of the ASK1-p38 pathway in aged kidney. To this end, we induced selective elimination of senescent cells, termed senolysis. Immunohistochemical analysis revealed that peritoneal injection of ABT-263, a widely used senolytic drug, reduced phospho-p38 signal in aged kidney (Fig. 5c), suggesting that senescent cells are involved in the activation of the ASK1-p38 pathway during renal aging.

**Fig. 6:**
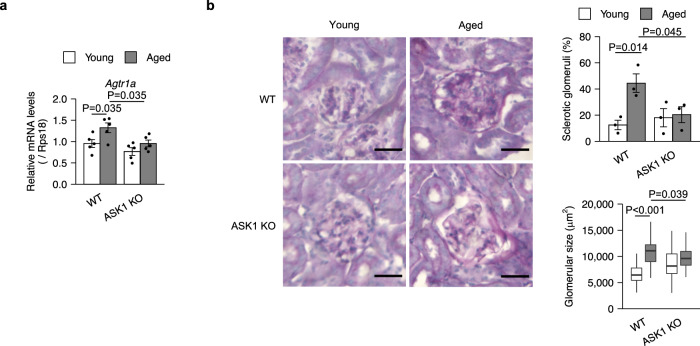
ASK1 contributes to age-related renal inflammation. **a** qPCR analysis of kidneys from young (3-month-old) and aged (20-month-old), wild-type (WT) and ASK1-knockout (ASK1 KO) mice. *n* = 5 mice per group. **b** Representative images of periodic acid-Schiff (PAS) staining (left). Percentages of sclerotic glomeruli (upper right) and quantification of glomerular size (lower right) of kidneys from young and aged, WT and ASK1 KO mice. Bars represent mean ± s.e.m. Center line, median; box limits, upper and lower quartiles; whiskers, 1.5× interquartile range. Scale bar, 50 μm. *n* = 3 mice per group. Statistical analysis was performed using two-way ANOVA followed by Dunnett’s multiple comparison test.

Glomerulosclerosis is a hallmark of age-related renal inflammation^39^. As a possible cause of glomerulosclerosis, it has been reported that senescent cells increase the expression of the angiotensin receptor Agtr1a in the kidney^8^. We confirmed that Agtr1a expression increased with age (Fig. 6a). This increase was impaired in ASK1 KO mice (Fig. 6a). Furthermore, age-related glomerular sclerosis, as indicated by periodic acid-Schiff (PAS) staining, and associated glomerular enlargement were ameliorated in ASK1 KO mice (Fig. 6b). Interestingly, the glomeruli of young ASK1 KO mice are slightly larger than those of young WT mice, possibly suggesting that ASK1 may also have an age-independent role in the kidney (Fig. 6b). These data suggest that ASK1 may contribute to age-related renal inflammation.

## Discussion

The SASP has been suggested as a contributor to age-associated inflammation and a potential therapeutic target for age-related diseases^19^. Previous studies have shown that pharmacological inhibition of NF-κB and other SASP drivers ameliorates ataxia, glomerulosclerosis, osteoporosis, and frailty^40–43^. However, until recently, there was no genetic evidence that the molecular mechanisms regulating the SASP are also involved in age-associated inflammation^44^. In the present study, we found that ASK1 is required for both the SASP and age-associated inflammation. In particular, ASK1 may contribute to age-related renal inflammation and glomerulosclerosis through the SASP. On the other hand, ASK1 appears to promote the elimination of oncogene-induced senescent hepatocytes through SASP-dependent macrophage recruitment, thereby preventing tumorigenesis. Thus, ASK1 is likely to play both positive and negative roles for the organism through the SASP, confirming the need for caution in using the SASP as a therapeutic target.

The role of ASK1 in senescence appears to depend on the senescence-inducing stimulus and the cell type. For example, our results suggest that ASK1 is involved in IL-6 expression during OIS but not during DIS in IMR-90 cells. It is unclear what causes this difference, but it is known that the expression levels of many SASP factors are higher in OIS than in DIS^45^. In addition, our results suggest that ASK1 is dispensable for p16 and p21 expression during OIS and DIS. This is in contrast to the role of ASK1 in adipocyte senescence induced by high-fat diet and LPS^46^.

Using hydrodynamic tail vein injection, we found that the elimination of oncogenic RAS-expressing hepatocytes is attenuated in ASK1 KO mice. This phenotype is rescued by re-expressing ASK1 along with oncogenic RAS. In hydrodynamic tail vein injection, genes are almost exclusively introduced into hepatocytes^47^. Therefore, the elimination of oncogenic RAS-expressing hepatocytes is likely to depend on ASK1 expressed in the hepatocytes themselves. However, we cannot completely exclude the possibility that ASK1 expressed in non-hepatic cells, such as immune cells, also contributes.

Using pathological mouse models, we have reported that ASK1 is involved in age-related diseases such as cardiac hypertrophy and Parkinson’s disease^26,27^. In addition, ASK1 has been implicated in osteoarthritis in aged mice^28^. Notably, these diseases have been reported to be ameliorated by senolysis and may therefore involve the ASK1-dependent SASP^29–31^. p38 has also been implicated in age-related diseases. In muscle stem cells, p38 is activated with age and decreases their proliferative capacity^48,49^. Furthermore, p38 is activated in aged and Alzheimer’s disease brains^50,51^. Therefore, chronic inflammation induced by the ASK1-p38 pathway may contribute to a variety of age-related diseases. Although ASK1 inhibition may increase the risk of certain cancers by impairing senescence surveillance, it has been well tolerated in clinical trials and may be a promising future therapeutic option for age-related diseases^52–54^.

## Methods

### Cell culture and treatments

IMR-90 normal human fibroblasts (ATCC, CCL-186) and HEK293T cells were cultured in DMEM (Sigma-Aldrich, D5796; Wako, 044-29765) supplemented with 10% FBS (BioWest, S1560-500) and 100 units/mL penicillin G (Meiji Seika). THP-1 human monocytes (RIKEN) were cultured in RPMI-1640 (Wako, 189-02025) supplemented with 10% FBS in a 5% CO_2_ atmosphere at 37 °C. For induction of the OIS, 4-hydroxy Tamoxifen (Cayman Chemical, 17308) at a final concentration of 200 nM was added to IMR-90 ER-KRAS cells. For induction of DIS, 200 ng/mL doxorubicin hydrochloride (Wako, 040-21521) were added to IMR-90 cells. For p38 inhibition experiments, 10 μM SB203580 (Tokyo Kasei, F0864) was added to the cells simultaneously with the induction of OIS and DIS.

### Lentivirus infection

For lentivirus production, HEK293T cells were transfected with the lentiviral vector pLenti-PGK-ER-KRAS^G12V^ (Addgene, 35635) and the packaging plasmids pCMV-VSV-G (Addgene, 8454) and psPAX2 (Addgene, 12260) using PEI MAX transfection reagent (Polyscience, 24765) for 24 h. The medium was replaced with 5 mL of fresh medium containing 1% bovine serum albumin (BSA, Iwai Chemical, A001) and collected 24 h later. This step was repeated. The collected media were combined and filtered through a 0.45 μm PVDF filter (Millipore, SLHVR33RS). IMR-90 cells were infected with lentiviruses in the presence of 8 μg/mL Polybrene (Nacalai Tesque, 17736-44) for 24 h, cultured in fresh media for 24 h, and selected with 100 μg/mL hygromycin (Nacalai Tesque, 07296-11) for 3 days.

### RNA isolation and qPCR

Total RNA was extracted using Isogen (Wako, 319-90211) and subjected to reverse transcription using a ReverTra Ace master mix (Toyobo, FSQ-301). qPCR was performed using a SYBR FAST qPCR kit (KAPA Biosystems, KK4602) and a QuantStudio 1 qPCR system (Applied Biosystems). The following primers were used: *GAPDH*, forward, 5′-AGCCACATCGCTCAGACAC-3′, reverse, 5′-GCCCAATACGACCAAATCC-3′; *ASK1*, forward, 5′-TGAGAAACCTAATGGAATCTTTAGC-3′, reverse, 5′-TGAGGGTTGTGATGTGTTCC-3′; *p16*, forward, 5′-CCAACGCACCGAATAGTTACG-3′, reverse, 5′-GCGCTGCCCATCATCATG-3′; *IL6*, forward, 5′-CCGGGAACGAAAGAGAAGCT-3′, reverse, 5′-GCGCTTGTGGAGAAGGAGTT-3′; *IL8*, forward, 5′-CTTTCCACCCCAAATTTATCAAAG-3′, reverse, 5′-CAGACAGAGCTCTCTTCCATCAGA-3′; *IL1B*, forward, 5′-TACCTGTCCTGCGTGTTGAA-3′, reverse, 5′-TCTTTGGGTAATTTTTGGGATCT-3′; *CCL2*, forward, 5′-AGTCTCTGCCGCCCTTCT-3′, reverse, 5′-GTGACTGGGGCATTGATTG-3′; *mRps18*, forward, 5′-TCCAGCACATTTTGCGAGTA-3′, reverse, 5′-CAGTGATGGCGAAGGCTATT-3′; *mCcl2*, forward, 5′-GTGGGGCGTTAACTGCAT-3′, reverse, 5′-CAGGTCCCTGTCATGCTTCT-3′; *mIl6*, forward, 5′-GCTACCAAACTGGATATAATCAGGA-3′, reverse, 5′-CCAGGTAGCTATGGTACTCCAGAA-3′; *mp21*, forward, 5′- CTGGTGATGTCCGACCTGTT-3′, reverse, 5′-TCAAAGTTCCACCGTTCTCG-3′; *mp16*, forward, 5′-CCCAACGCCCCGAACT-3′, reverse, 5′-GCAGAAGAGCTGCTACGTGAA-3′; *mIl1b*, forward, 5′-AAGAGCTTCAGGCAGGCAGTATCA-3′, reverse, 5′-ATGAGTCACAGAGGATGGGCTCTT-3′; *mCxcl1*, forward, 5′-CCCGCTCGCTTCTCTGT-3′, reverse, 5′-CTTTTGGACAATTTTCTGAACCAAG-3′; *mCxcl2*, forward, 5′-CACCAACCACCAGGCTACAG-3′, reverse, 5′-GCTTCAGGGTCAAGGCAAAC-3′; *mAgtr1a*, forward, 5′-AAGGGCCATTTTGCTTTTCT-3′, reverse, 5′-AACTCACAGCAACCCTCCAA-3′.

Transcript levels were normalized to *GAPDH* (human) and *mRps18* (mouse).

### siRNA transfection

siRNAs with the following target sequences were purchased from Dharmacon: NT #1 (hereafter ON-TARGETplus), 5′-UGGUUUACAUGUCGACUAA-3′; ASK1 #1 5′-GGGAAUCUAUACUCAAUGA-3′; ASK1 #2 5′-ACACUACAGUCAGGAAUUA-3′. For the knockdown of MAP3Ks, siRNAs (ON-TARGETplus SMARTpool) targeting each MAP3Ks were purchased from Dharmacon. siRNA transfection was performed at a final concentration of 10 nM using Lipofectamine RNAiMAX transfection reagent (Invitrogen, 13778500) and Opti-MEM (Gibco, 31985070).

### Immunoblotting

Cells were lysed in RIPA buffer (50 mM Tris-HCl [pH 8.0], 150 mM NaCl, 1% NP-40, 0.5% sodium deoxycholate, 0.1% sodium dodecyl sulfate [SDS], 1 mM phenylmethylsulfonyl fluoride, 5 μg/mL leupeptin, 8 mM NaF, 12 mM β-glycerophosphate, 1 mM Na_3_VO_4_, 1.2 mM Na_2_MoO_4_, 5 μM cantharidin, 2 mM imidazole). Lysates were clarified by centrifugation at 17,500 × *g* for 10 min at 4 °C. The protein concentrations of the lysates were quantified using a BCA protein assay kit (Wako, 297-73101) and equalized. The lysates were then mixed with 2 × SDS sample buffer (125 mM Tris-HCl [pH 6.8], 4% SDS, 20% glycerol, 200 μg/mL bromophenol blue, 10% β-mercaptoethanol) and heated at 98 °C for 3 min. SDS samples were subjected to SDS-polyacrylamide gel electrophoresis (SDS-PAGE) and transferred to Immobilon-P membranes (Millipore, IPVH00010). The membranes were blocked with 5% skim milk (Megmilk Snow Brand) in TBS-T (50 mM Tris-HCl [pH8.0], 150 mM NaCl, 0.05% Tween 20) for 30 min at room temperature and incubated with primary antibodies in TBS- T containing 5% BSA and 0.1% NaN_3_ overnight at 4 °C. The membranes were then incubated with secondary antibodies in TBS-T containing 5% skim milk for 2 h at room temperature, followed by detection using ECL Select detection reagent (Amersham, RPN2235) and a FUSION Solo S chemiluminescence imaging system (Vilber) or X-ray films (FUJIFILM, 47410-22167 or 47410-26615). The following primary antibodies were used: anti-ASK1 (1:1000, Abcam, ab45178), anti-phospho-p38 (1:5000, Cell Signaling, #4511), anti-p38α (1:1000, Cell Signaling, #9228), anti-p16 (1:1000, Abcam, ab108349), anti-KRAS (1:1000, Santa Cruz Biotechnology, sc-30), anti-α-tubulin (1:10,000, Bio-Rad, MCA77G). Anti-phospho-ASK antibody (1:1000) was generated as previously described^55^.

### Transwell migration assay

To generate CM, IMR-90 ER-KRAS cells were treated with or without 4OHT for 8 days and then reseeded. The next day, the cells were washed with PBS and then cultured in FBS-free RPMI-1640 for 3 days. Migration assays were performed using transwell polycarbonate membrane inserts with 8 μm pore diameter (FALCON, 353097). 750 μL of CM was added to the bottom chambers. THP-1 cells (5 × 10^5^ cells) resuspended in FBS-free RPMI-1640 medium were added to the inserts. THP-1 cells were allowed to migrate into the bottom chamber for 6 h at 37 °C and the number of cells that had migrated to the bottom chamber was counted. For CCL2 neutralization experiments, 3 μg/mL mouse anti-CCL2 (R&D Systems, 23007) or mouse anti-IgG (Santa Cruz Biotechnology, sc-2025) was added to CM.

### ELISA

To measure the secreted CCL2 levels in CM, ELISA was performed using Human MCP-1 ELISA kit (Proteintech, KE00277) following manufacture’s protocol.

### Mice

WT and ASK1 KO mice on the C57BL/6 background were described previously^56^. Mice were maintained in a specific pathogen-free facility and age-matched mice were used for the experiment. All the experiments were performed following the experimental protocol approved by the animal ethics committee of the University of Tokyo. We have complied with all relevant ethical regulations for animal use. Male C57BL/6J mice were used.

### Plasmids

To generate pT/Caggs-NRASV12/D38A, a point mutation was introduced into pT/Caggs-NRASV12 (Addgene, #20205) by PCR using a PrimeSTAR Mutagenesis Basal Kit (Takara, R046A) and the following primers; forward, 5′-ATAGAGGCTTCTTACAGAAAACAAGTG-3′, reverse, 5′-GTAAGAAGCCTCTATGGTGGGATCATA-3′.

### Hydrodynamic gene delivery

Mice aged 8–10 weeks of age were subjected to hydrodynamic injection. 25 μg of pT/Caggs-NRASV12 or pT/Caggs-NRASV12/D38A and 5 μg of PT2/c-Luc//PGK-SB-13 (Addgene, 20207) were suspended in 0.9% saline solution at a final volume of 10% of the body weight and injected via the tail vein within 8 s. Vectors were prepared using a QIAGEN EndoFree Mega Kit (QIAGEN).

### Immunohistochemistry

Mouse tissues were perfused and immersed O/N in 4% paraformaldehyde/PBS for fixation, then cleared with 20% sucrose solution. Fixed tissues were embedded in CryoMount I (Muto PureChemicals) and 8 μm-thick sections were cut using a cryostat (Leica). For immunofluorescence assay, sections were blocked in 3% BSA/PBS for 30 min at room temperature, and then incubated with anti-F4/80 (1:100, Abcam, ab6640), anti-CD4 (1:100, Abcam, ab183685), anti-CD8 (1:100, Abcam, ab217344), anti-CD163 (1:100, Proteintech, 16646-1-AP), and anti-phospho-p38 (1:100, Cell Signaling, #4511) in 3% BSA/PBS for O/N at 4 °C after antigen retrieval. Antigen retrieval was carried out with sodium citrate buffer (pH 6.0) for 20 min at 92 °C. Then the sections were incubated with Alexa Fluor secondary antibodies (1:100, Invitrogen) and Hoechst 33342 (1:1000) for 2 h at room temperature. Images were acquired using a TCS SP5 confocal microscope (Leica) with 40 × 1.25 NA and 63 × 1.4 NA oil immersion objectives (Leica). Periodic acid-Schiff staining was performed using a PAS staining kit (Sigma-Aldrich, 101646) according to the manufacturer’s instructions. Kidney sections were then counterstained with hematoxylin (Wako, 131-09665).

### Luciferase activity assay

Mouse livers were homogenized in Luciferase Culture Lysis Reagent (Promega). Lysates were clarified by centrifugation at 13,000 × *g* for 20 min at 4 °C. Supernatants were collected and analyzed using a Luciferase Assay System (Promega). Luminescence was measured using a Varioskan Flash (Thermo Fisher Scientific).

### TCGA data analyses

For TCGA analyses, RNA-seq datasets were obtained from cBioportal (http://www.cbioportal.org). For a given cancer type, the expression level of each gene was normalized to TPM values and ASK1 expression levels were compared between normal and tumor samples. Tumor samples were ranked based on ASK1 expression levels and were evenly divided into four groups. Statistical comparisons were performed between the first group (samples with the lowest 25% expression) and the last group (samples with the highest 25% expression) for inflammatory genes or GAPDH. Survival was determined using the Kaplan-Meier method.

### Senolysis

ABT-263 was dissolved in DMSO and then diluted to create a working solution (30% propylene glycol, 5% TWEEN 80, 3.3% dextrose in water, pH 4–5). For the selective elimination of senescent cells in mice, 50 mg/kg body weight of ABT-263 (Chemietek, CT-A263) or DMSO was injected intraperitoneally for 2 consecutive days. Mice were sacrificed 5 days after the last treatment.

### Statistics and reproducibility

R software was used for statistical analysis. After evaluation of normality using Shapiro-Wilk test, statistical differences were measured using the methods indicated in the figure legends. P < 0.05 was considered statistically significant. No statistical method was used to predetermine sample size. Results were replicated in independent experiments. Number of biological replicates is given in legends.

### Reporting summary

Further information on research design is available in the Nature Portfolio Reporting Summary linked to this article.

### Supplementary information

Supplementary information

Description of Additional Supplementary Files

Supplementary Data 1

Reporting Summary

## Data Availability

All data supporting the findings of this study are available within the paper and its Supplementary Information. Uncropped and unedited blot images are provided in Supplementary Fig. 3. All source data for the graphs in this study are provided in Supplementary Data 1. Newly generated plasmids were deposited at Addgene (pT/Caggs-NRAS G12V/D38A: 221073, pT/Caggs-NRAS G12V-IRES-ASK1: 221074).
