# Supplementary material for: Apoptosis signal-regulating kinase 1 promotes inflammation in senescence and aging
Source: Commun Biol. 2024 Jun 5;7:691. doi: 10.1038/s42003-024-06386-0 (PMC11153534; doi:10.1038/s42003-024-06386-0)
Supplement: Supplementary file 1 — Supplementary information [file 42003_2024_6386_MOESM1_ESM.pdf]

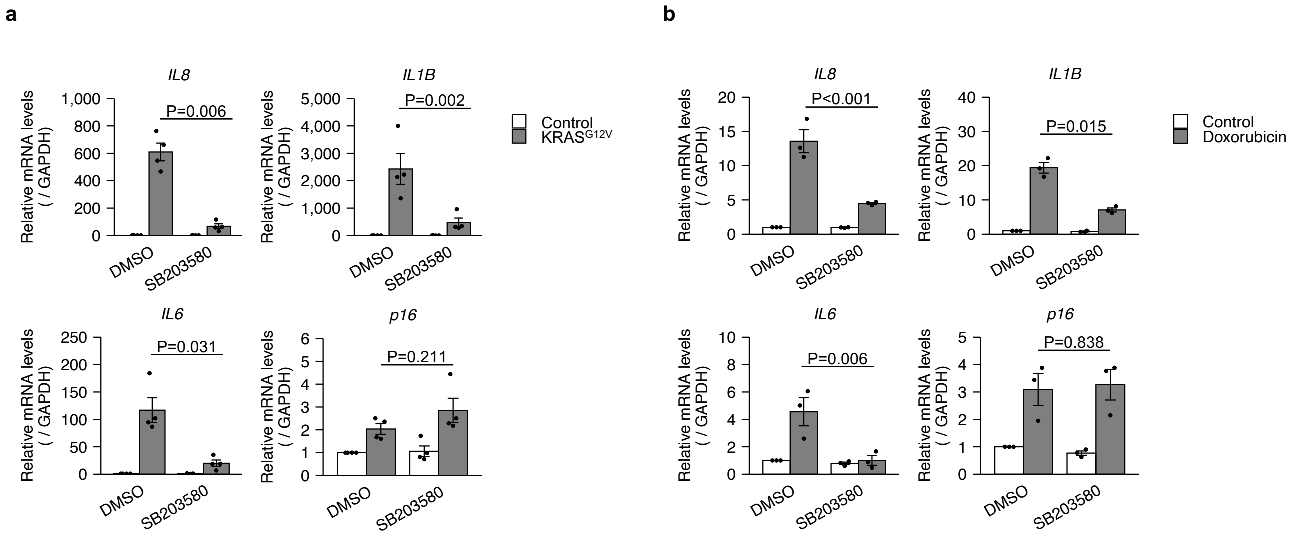

Supplementary Fig. 1|p38 inhibition suppresses the expression of SASP factors but not p16. a, qPCR analysis of IMR-90 ER-KRAS cells treated with 4OHT and SB203580 for 12 days. $n = 4$ independent experiments. **b,** qPCR analysis of IMR-90 cells treated with doxorubicin and SB203580 for 12 days. $n = 3$ independent experiments. Bars represent mean $\pm$ s.e.m. (**a, b**). Statistical analysis was performed using unpaired two-tailed Student's t-test (**a, b**).

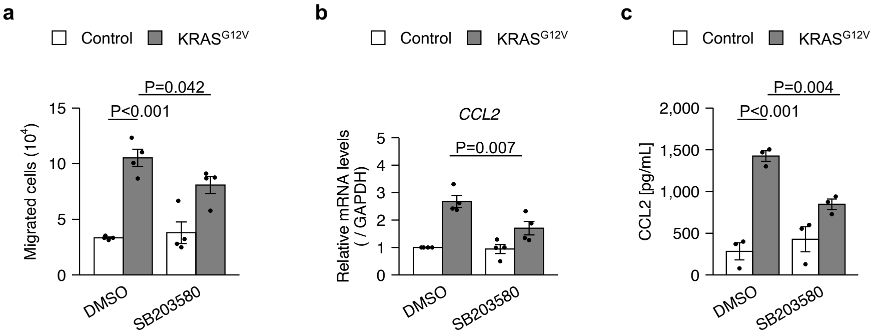

Supplementary Fig. 2|p38 inhibition suppresses CCL2-mediated migration of THP-1 cells. a, Migration assay of THP-1 cells. CM was collected from proliferative and senescent IMR-90 ER-KRAS cells treated with SB203580. *n* = 4 independent experiments. **b, c,** qPCR analysis (**b**) and ELISA (**c**) of IMR-90 ER-KRAS cells treated with 4OHT and SB203580 for 12 days. *n* = 4 (**b**), 3 (**c**) independent experiments. Bars represent mean ± s.e.m. (**a-c**). Statistical analysis was performed using two-way ANOVA followed by Dunnett's multiple comparison test (**a, c**) and unpaired two-tailed Student's t-test (**b**).

Related to Fig. 1e

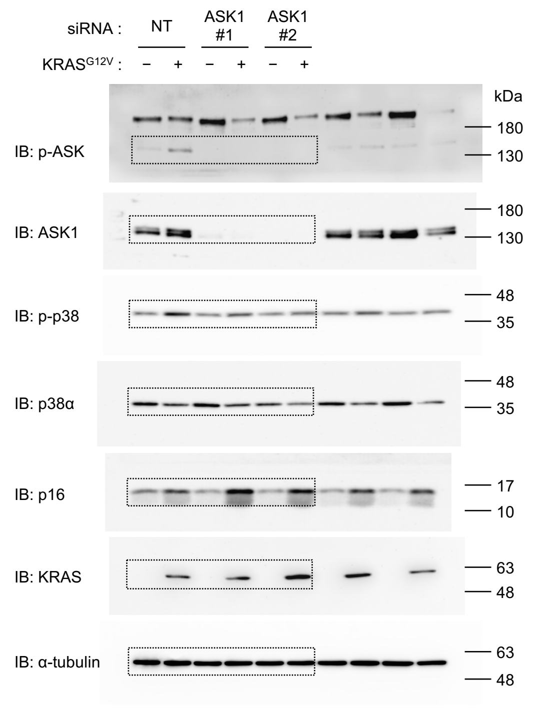

Related to Fig. 1f

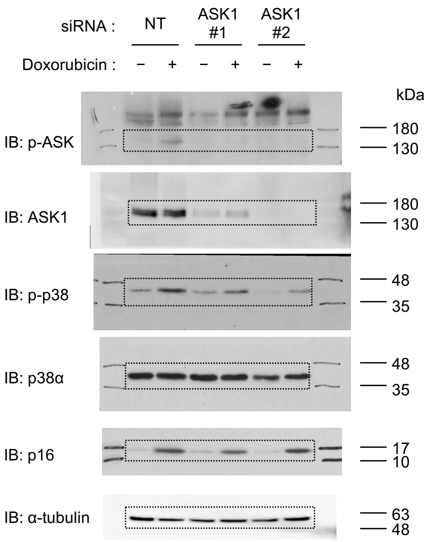

Related to Fig. 5a

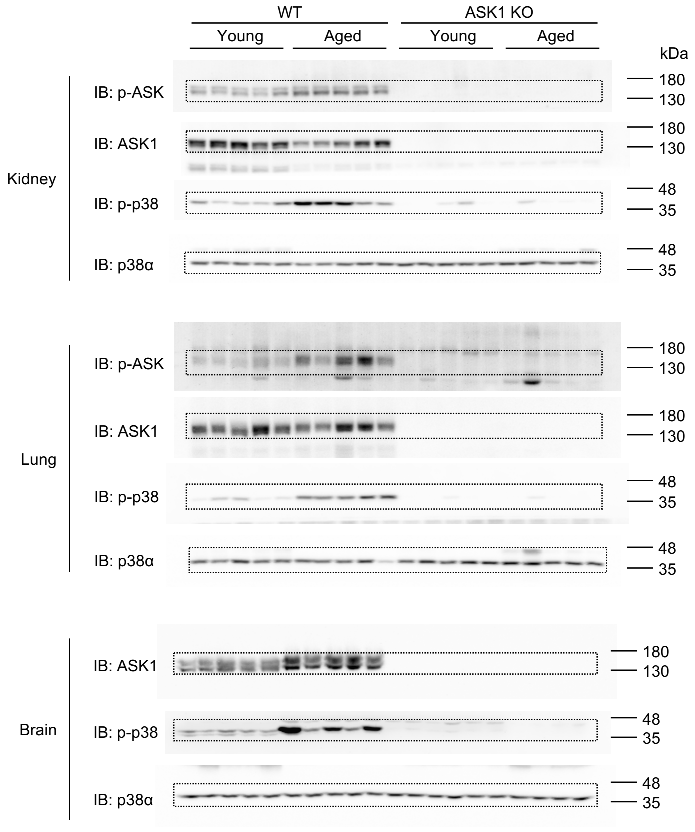

Supplementary Fig. 3 | Uncropped blot images
