# Supplementary material for: Apoptosis signal-regulating kinase 1 promotes inflammation in senescence and aging
Source: Commun Biol. 2024 Jun 5;7:691. doi: 10.1038/s42003-024-06386-0 (PMC11153534; doi:10.1038/s42003-024-06386-0)
Supplement: Supplementary file 4 — Reporting Summary [file 42003_2024_6386_MOESM4_ESM.pdf]

nature portfolio

Corresponding author(s): Shota Yamauchi and Hidenori Ichijo

Last updated by author(s): May 13, 2024

Reporting Summary

Nature Portfolio wishes to improve the reproducibility of the work that we publish. This form provides structure for consistency and transparency in reporting. For further information on Nature Portfolio policies, see our Editorial Policies and the Editorial Policy Checklist.

Statistics

For all statistical analyses, confirm that the following items are present in the figure legend, table legend, main text, or Methods section.

n/a	Confirmed	
☐	☒	The exact sample size (*n*) for each experimental group/condition, given as a discrete number and unit of measurement
☐	☒	A statement on whether measurements were taken from distinct samples or whether the same sample was measured repeatedly
☐	☒	The statistical test(s) used AND whether they are one- or two-sided<br>*Only common tests should be described solely by name; describe more complex techniques in the Methods section.*
☒	☐	A description of all covariates tested
☐	☒	A description of any assumptions or corrections, such as tests of normality and adjustment for multiple comparisons
☐	☒	A full description of the statistical parameters including central tendency (e.g. means) or other basic estimates (e.g. regression coefficient) AND variation (e.g. standard deviation) or associated estimates of uncertainty (e.g. confidence intervals)
☐	☒	For null hypothesis testing, the test statistic (e.g. *F*, *t*, *r*) with confidence intervals, effect sizes, degrees of freedom and *P* value noted<br>*Give P values as exact values whenever suitable.*
☒	☐	For Bayesian analysis, information on the choice of priors and Markov chain Monte Carlo settings
☒	☐	For hierarchical and complex designs, identification of the appropriate level for tests and full reporting of outcomes
☒	☐	Estimates of effect sizes (e.g. Cohen's *d*, Pearson's *r*), indicating how they were calculated

Our web collection on statistics for biologists contains articles on many of the points above.

Software and code

Policy information about availability of computer code

Data collection	Fusion Edge (Vilber) software for immunoblotting data and Leica Application Suite software for microscopy data
Data analysis	R version 4.0.5, RStudio version 1.4.1106, and Microsoft Excel version 16.65 for statistical analysis; ApE version 2.0.61 for sequence data

For manuscripts utilizing custom algorithms or software that are central to the research but not yet described in published literature, software must be made available to editors and reviewers. We strongly encourage code deposition in a community repository (e.g. GitHub). See the Nature Portfolio guidelines for submitting code & software for further information.

Data

Policy information about availability of data

All manuscripts must include a data availability statement. This statement should provide the following information, where applicable:

- Accession codes, unique identifiers, or web links for publicly available datasets
- A description of any restrictions on data availability
- For clinical datasets or third party data, please ensure that the statement adheres to our policy

All data supporting the findings of this study are available within the paper and its Supplementary Information. Uncropped and unedited blot images are provided in Supplementary Fig. 3. All source data for the graphs in this study are provided in Supplementary Data 1.

Research involving human participants, their data, or biological material

Policy information about studies with human participants or human data. See also policy information about sex, gender (identity/presentation), and sexual orientation and race, ethnicity and racism.

Reporting on sex and gender	The study did not involve human participants, their data, or biological material.
Reporting on race, ethnicity, or other socially relevant groupings	The study did not involve human participants, their data, or biological material.
Population characteristics	The study did not involve human participants, their data, or biological material.
Recruitment	The study did not involve human participants, their data, or biological material.
Ethics oversight	The study did not involve human participants, their data, or biological material.

Note that full information on the approval of the study protocol must also be provided in the manuscript.

Field-specific reporting

Please select the one below that is the best fit for your research. If you are not sure, read the appropriate sections before making your selection.

☒ Life sciences ☐ Behavioural & social sciences ☐ Ecological, evolutionary & environmental sciences

For a reference copy of the document with all sections, see nature.com/documents/nr-reporting-summary-flat.pdf

Life sciences study design

All studies must disclose on these points even when the disclosure is negative.

Sample size	No statistical method was used to predetermine the sample size.
Data exclusions	No data were excluded from the analyses.
Replication	Results were replicated in independent experiments. Number of biological replicates is given in legends.
Randomization	Mice of the same age were randomly assigned to experimental and control groups.
Blinding	No blinding was performed.

Reporting for specific materials, systems and methods

We require information from authors about some types of materials, experimental systems and methods used in many studies. Here, indicate whether each material, system or method listed is relevant to your study. If you are not sure if a list item applies to your research, read the appropriate section before selecting a response.

Materials & experimental systems

n/a	Involved in the study	
☐	☒	Antibodies
☐	☒	Eukaryotic cell lines
☒	☐	Palaeontology and archaeology
☐	☒	Animals and other organisms
☒	☐	Clinical data
☒	☐	Dual use research of concern
☒	☐	Plants

Methods

n/a	Involved in the study	
☒	☐	ChIP-seq
☒	☐	Flow cytometry
☒	☐	MRI-based neuroimaging

Antibodies

Antibodies used	Anti-p16 (Abcam, ab108349), anti-alpha-tubulin (Bio-Rad, MCA77G), anti-KRAS (Santa Cruz Biotechnology, sc-30), anti-F4/80 (Abcam, ab6640), anti-CD4 (Abcam, ab183685), anti-CD8 (Abcam, ab217344), anti-CD163 (Proteintech, 16646-1-AP), anti-CCL2 (R&D Systems, 23007), anti-Control IgG (Santa-Cruz Biotechnology, sc-2025), anti-phospho-p38 (Cell Signaling, #4511), anti-p38-alpha (Cell Signaling, #9228), anti-ASK1(Abcam, ab45178), anti-phospho-ASK (Tobiume et al., J. Cell. Physiol. 2002) antibodies were used.

Validation	Anti-ASK1 antibodies were validated by siRNA knockdown. Anti-p16 (Abcam), anti-KRAS, anti-phospho-ASK antibodies were validated by stimulating cells. Other antibodies were validated by the manufacturers.

Eukaryotic cell lines

Policy information about cell lines and Sex and Gender in Research

Cell line source(s)	IMR-90 cells and HEK293T cells were from ATCC. THP-1 cells were from RIKEN.
Authentication	Cell lines were not externally authenticated.
Mycoplasma contamination	Cell lines were tested negative for mycoplasma contamination by staining.
Commonly misidentified lines (See ICLAC register)	No commonly misidentified lines were used in this study.

Animals and other research organisms

Policy information about studies involving animals; ARRIVE guidelines recommended for reporting animal research, and Sex and Gender in Research

Laboratory animals	C57BL/6J mice aged 8–10 weeks were used unless otherwise indicated. Mice were group housed under a 12:12 h light/dark cycle with free access to food and water.
Wild animals	The study did not involve wild animals.
Reporting on sex	Male mice were used.
Field-collected samples	The study did not involve samples collected from the field.
Ethics oversight	All the experiments were performed following the experimental protocol approved by the animal ethics committee of the University of Tokyo. We have complied with all relevant ethical regulations for animal use. Male C57BL/6J mice were used.

Note that full information on the approval of the study protocol must also be provided in the manuscript.

Plants

Seed stocks	N/A
Novel plant genotypes	N/A
Authentication	N/A
